# Association of Glutathione s-transferase M1 and T1 gene polymorphisms with the susceptibility to acquired sensorineural hearing loss: a systematic review and meta-analysis

**DOI:** 10.1038/s41598-018-37386-w

**Published:** 2019-01-29

**Authors:** Shimin Zong, Xue Zeng, Yexiao Guan, Tianyi Liu, Pan Luo, Fangmin Wan, Yanji Qu, Pei Chen, Hongjun Xiao

**Affiliations:** 1 Department of Otorhinolaryngology, Union Hospital, Tongji Medical College, Huazhong University of Science and Technology, Wuhan, 430022 China; 2 Department of Obstetrics and Gynecology, Tongji Hospital, Tongji Medical College, Huazhong University of Science and Technology, Wuhan, 430032 China; 3 Department of Otorhinolaryngology, Wuhan Integrated TCM and Western Medicine Hospital (Wuhan No. 1 hospital), Tongji Medical College, Huazhong University of Science and Technology, Wuhan, 430022 China

## Abstract

Acquired sensorineural hearing loss (SNHL), including age-related hearing loss (ARHL), noise-induced hearing loss (NIHL), drug-induced hearing loss (DIHL) and sudden sensorineural hearing loss (SSHL), is one of the most common sensory deficits in humans. Several studies have reported that antioxidant gene glutathione s-transferase M1 and T1 (GST M1 and T1) polymorphisms have a close relationship with the susceptibility to acquired SNHL, but other articles have reported opposite results. This meta-analysis aims to identify whether an association exists between GST M1 and T1 polymorphisms and the susceptibility to acquired SNHL. Seventeen independent studies containing 1749 cases and 2018 controls were included. According to the I^2^ value of the heterogeneity test, random-effects model was selected to calculate the pooled odds ratios (ORs) with their 95% confidence intervals (95% CIs) and p values. The pooled ORs (95% CI, p-value) of GST M1 and T1 were 1.186(0.955–1.473, p = 0.122) and 1.107(0.841–1.458, p = 1.467), respectively. In addition, subgroup analyses according to the type of SNHL and ethnicity showed no relationship between GST M1 and T1 polymorphisms and the susceptibility to acquired SNHL. Our results suggest that no significant relationship was found between GST M1 and T1 polymorphisms and the susceptibility to acquired SNHL.

## Introduction

Acquired sensorineural hearing loss (SNHL), including age-related hearing loss (ARHL), noise-induced hearing loss (NIHL), drug-induced hearing loss (DIHL) and sudden sensorineural hearing loss (SSHL), is one of the most common sensory deficits in humans in modern society^1^. Approximately 360 million people worldwide suffer from this health problem^2^. People with acquired SNHL exhibit decreased hearing sensitivity and a decline in speech intelligibility, which can lead to serious difficulties in an individual’s communication and social interactions and consequently reduce life expectancy^3,4^. Despite the high prevalence and serious effects of acquired SNHL, few therapeutic methods have been found to be clinically effective^5^.

The susceptibility to acquired SNHL among individuals is diverse. Some individuals are more susceptible to acquired SNHL, while others are not. Several studies have suggested that this individual difference in susceptibility to acquired SNHL is mostly due to the different genetic backgrounds of individuals, especially genetic polymorphisms that affect the expression of some functional proteins or enzymes^6–10^. Therefore, exploring these genetic differences and then exploiting them may enable the development of individual prevention strategies for SNHL.

Oxidative stress has been proven to be the most important molecular mechanism in the pathogenesis of acquired SNHL^11–14^. Researchers have successfully alleviated several kinds of SNHL with the application of antioxidants in animal experiments^11,13–15^. Glutathione s-transferase (GST) encodes a system of antioxidative enzymes that have been demonstrated to play an important role in antioxidative protection in cochlear cells^16–18^. Among the GST subclasses, GST T1 and M1 are genetically deleted (null genotype) in a high percentage in humans. Approximately 30–50% of individuals have a null genotype for GST M1, depending on their race^19^, and 25–40% carry the null genotype of GST T1^20^. Rabinnowitz *et al*. once suggested that individuals with the null genotypes of GST M1 or GST T1 are more susceptible to oxidative stress damage and are possibly more susceptible to NIHL^21^.

Many studies have attempted to correlate mutant genotypes of GST to the susceptibility to acquired SNHL. Some have demonstrated a close relationship between GST M1 or T1 polymorphisms and the susceptibility to SNHL^6,22–25^, and others have reported conflicting results^8,10,16,26–34^. Meta-analysis is an effective way to address this type of contradiction. Therefore, we performed this meta-analysis to identify whether a close association exists between GST M1 and T1 polymorphisms and the susceptibility to acquired SNHL and whether GST M1 and T1 polymorphisms can serve as predictive factors for the susceptibility to acquired SNHL.

## Results

### Literature search and characteristics of the included studies

**Figure 1:**
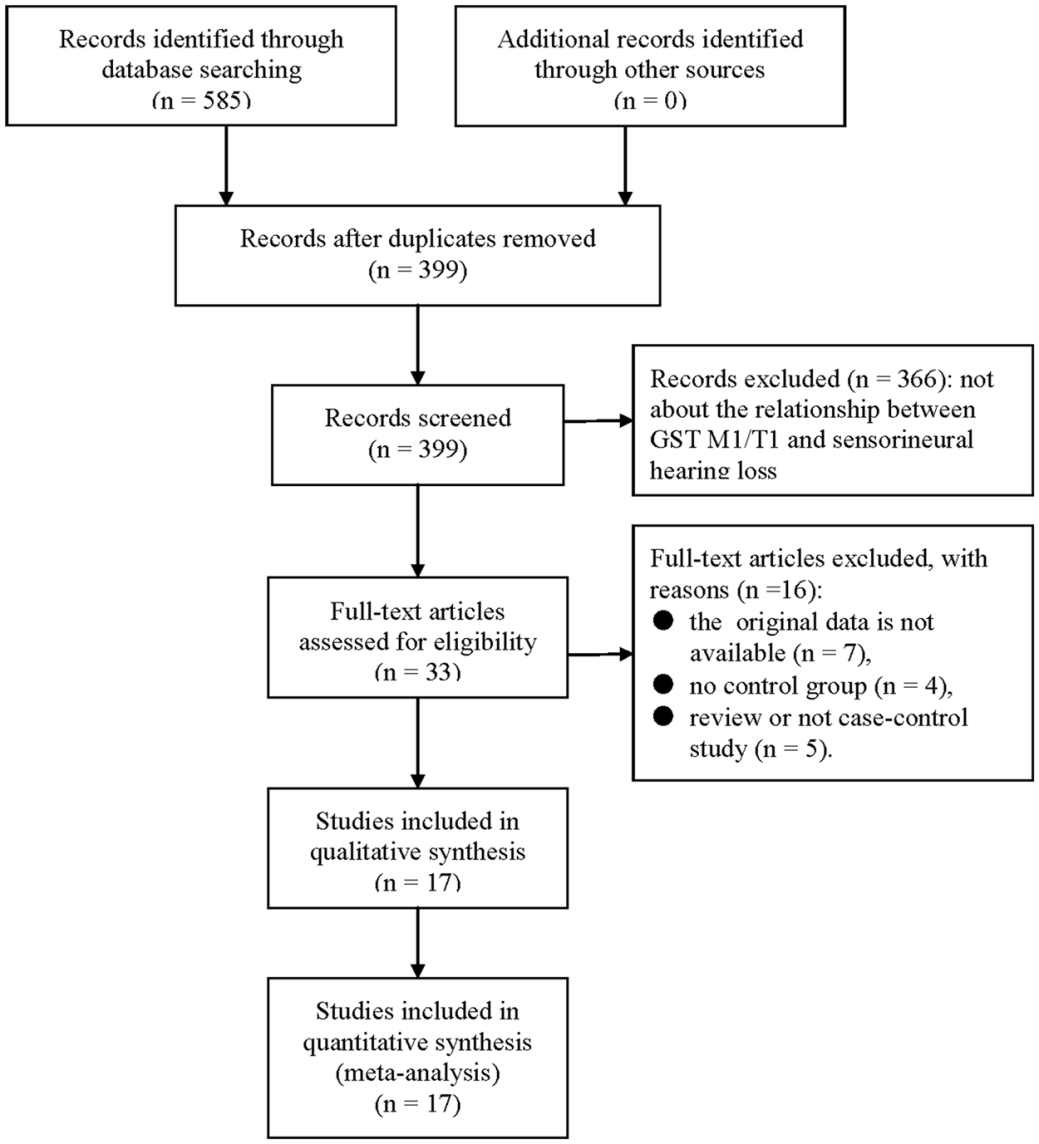
Flow diagram of the study selection process.

The literature selection process is shown in Fig. 1. Through the search in the databases, 585 potentially relevant records were identified, 399 of which were retained after duplicates were removed. After screening the records, 366 records were excluded because they did not discuss the relationship between GST M1 and T1 polymorphisms and acquired SNHL. The remaining 33 articles were assessed for eligibility via full-text screening. Of these, 16 studies were excluded for various reasons, such as unavailable original data, no control groups, reviews or non-original articles. Finally, 17 independent studies were included in the meta-analysis. Therefore, a total of 1749 cases with acquired SNHL and 2018 controls were included. Table 1 summarizes the basic information of the 17 included eligible studies.

**Table 1:** Characteristics of included studies.

First author	Year	Country	Ethnicity	Genotype method	NOS score	Hearing loss type	Case	Control	GST T1				GST M1			
									Case		Control		Case		Control	
									WT	null	WT	null	WT	null	WT	null
Manche	2016	India	Indian	Multiplex-PCR	8	ARHL	220	270	119	101	202	68	121	99	201	69
Zhu	2011	China	Chinese	Multiplex-PCR	8	ARHL	110	114	61	49	58	56	39	71	33	81
Bared	2010	USA	Mixed	Multiplex-PCR	8	ARHL	55	79	21	31	52	27	16	36	41	38
Ates	2005	Turkey	Turkish	Real-time PCR	8	ARHL	68	69	54	14	53	16	36	32	40	29
Shen	2012	China	Chinese	PCR-RFLP	8	NIHL	444	445	215	229	210	235	198	246	253	192
Abreu-Silva	2011	Brazil	Brazilian	PCR-RFLP	8	NIHL	151	104	40	111	22	82	115	36	77	27
Liu	2006	China	Chinese	PCR	8	NIHL	123	123	58	60	48	66	38	85	54	69
Yang	2005	China	Chinese	Multiplex-PCR	8	NIHL	93	101	31	62	47	57	37	56	35	66
Carlsson	2005	Sweden	Swedish	PCR-RFLP	8	NIHL	103	112	90	13	104	8	50	53	59	53
Choeyprasert	2013	Thailand	German	Multiplex-PCR	8	DIHL	55	13	38	17	4	9	24	31	5	8
Jurajda	2012	Czech	Czechs	PCR	7	DIHL	12	26	10	2	20	6	4	8	10	16
Palodetto	2010	Brazil	Brazilian	Multiplex-PCR	5	DIHL	10	20	8	2	13	7	6	4	11	9
Barahmani	2009	USA	Mixed	Multiplex-PCR	7	DIHL	19	15	13	6	10	5	11	8	9	6
Oldenburg	2007	Norway	Norwegians	Multiplex-PCR	7	DIHL	89	84	75	14	70	14	49	40	45	39
Peters	2000	Germany	Thai	PCR	8	DIHL	19	20	12	8	16	3	11	9	8	11
Um	2011	Korea	Korean	Multiplex-PCR	6	SSHL	98	343	51	47	173	170	40	58	138	205
Cadoni	2006	Italy	Italian	Multiplex-PCR	8	SSHL	80	80	60	20	62	18	41	39	36	44

NOS: Newcastle-Ottawa Scale. GST: glutathione s-transferase. ARHL: age-related hearing loss. NIHL: noise-induced hearing loss. DIHL: drug-induced hearing loss. SSHL: sudden sensorineural hearing loss. WT: wild type. PCR-RFLP: polymerase chain reaction-restriction fragment length polymorphis.

### The relationship between GST M1 and T1 polymorphisms and the susceptibility to acquired sensorineural hearing loss

**Figure 2:**
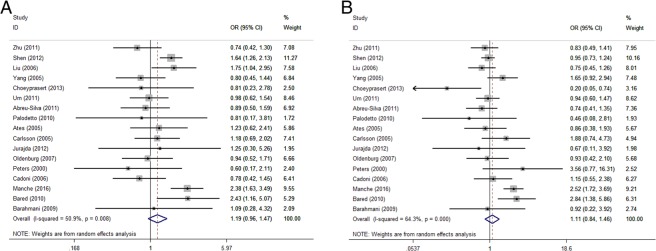
Forest plot presenting the association between GST M1 (**A**) and T1 (**B**) polymorphisms and the susceptibility to acquired SNHL.

The I^2^ values for GST M1 and T1 were 50.9% and 64.3%, respectively, which are shown in Fig. 2. Both I^2^ values were ≥30%, so we used a random-effects model to calculate the pooled odds ratios (ORs) and 95% confidence intervals (95% CIs). The pooled ORs (95% CI, p-value) of GST M1 and T1 were 1.186(0.955–1.473, 0.122) and 1.107(0.841–1.458, 1.467), respectively.

**Figure 3:**
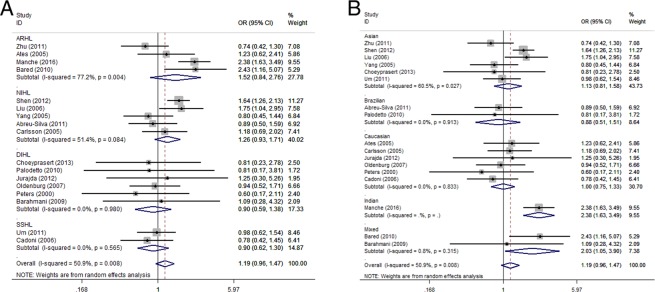
Subgroup analysis of the association between GST M1 and the susceptibility to acquired SNHL according to the acquired SNHL types (**A**) and ethnicity (**B**).

To address heterogeneity, we performed a subgroup analysis according to the acquired SNHL type and ethnicity. For GST M1, no heterogeneity was observed in the DIHL and the SSHL subgroups. However, relatively strong heterogeneity was observed in the ARHL and NIHL subgroups (77.2% for the ARHL subgroup and 51.4% for the NIHL subgroup, Fig. 3A). For the subgroup analysis according to ethnicity, no heterogeneity was observed in the Caucasian subgroup, while intermediate heterogeneity was observed in the Asian subgroup (49.7%, Fig. 3B). In addition, no statistically significant relationship between GST M1 polymorphisms and the susceptibility to acquired SNHL was found in any of the acquired SNHL type and ethnicity subgroups.

**Figure 4:**
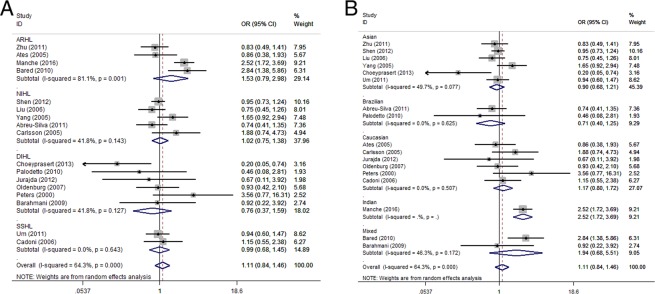
Subgroup analysis of the association between GST T1 and the susceptibility to acquired SNHL according to the acquired SNHL types (**A**) and ethnicity (**B**).

As exhibited in Fig. 4, the subgroup analysis according to the type of acquired SNHL and ethnicity of GST T1 showed similar results to those of GST M1.

### Sensitivity analysis

**Figure 5:**
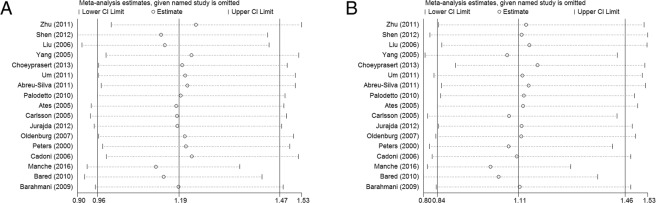
Sensitivity analysis of the pooled effect size on the association between GST M1 (**A**) and T1 (**B**) polymorphisms and the susceptibility to acquired SNHL.

The included studies were removed one by one to investigate whether the study removed was the source of heterogeneity. Figure 5 shows that there was no significant difference in the pooled effect size when any of the studies were excluded. This result of the sensitivity analysis demonstrates that the pooled effect size of this meta-analysis was stable.

### Publication bias

**Figure 6:**
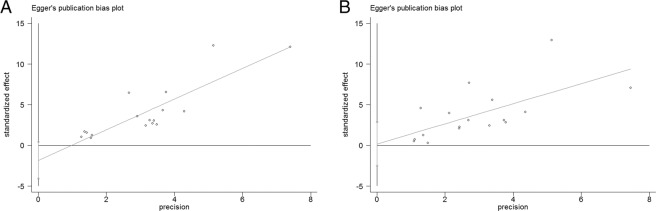
Publication bias analyses (Egger’s test) for the pooled effect size.

The risk of publication bias was analyzed by Egger’s test. The results are shown in Fig. 6. The p-values (95% CI) of GST M1 and T1 were 0.102(−4.071, 0.410) and 0.887(−2.504, 2.870), respectively. Both p values were >0.05, and the 95% CIs contained 0. Therefore, no publication bias was considered.

## Discussion

Oxidative stress is the most important and common molecular mechanism of acquired SNHL^11–14^. However, difficulties remain with the effective clinical application of antioxidants^11,13,15,35^. Therefore, identifying susceptibility factors of individuals in terms of the oxidative stress-related genetic background or gene polymorphisms may represent a new concept. GST M1 and T1 have been found to be important antioxidant enzymes in the human body, and they are associated with several kinds of oxidative stress-related diseases, including acquired SNHL^6,22–25,36^. However, in previous studies on the association of GST M1 and T1 polymorphisms with the susceptibility to acquired SNHL, the results are inconsistent or even contradictory. There may be at least two reasons for this inconsistency: (1) most of the previous studies were single-center studies with small sample sizes; and (2) most of the previous studies focused on the associations of GST M1 and T1 polymorphisms with only one type of acquired SNHL and neglected the fact that oxidative stress is the most important and common molecular mechanism of acquired SNHL. Based on the above theoretical basis, this meta-analysis included more studies on oxidative stress-related acquired SNHL types and larger sample sizes to further identify the association of GST M1 and T1 polymorphisms with the susceptibility to acquired SNHL. According to our results, neither the collective nor the subgroup analyses suggested an association between GST M1 and T1 polymorphisms and susceptibility to acquired SNHL.

A meta-analysis regarding the association between GST M1 and T1 polymorphisms and NIHL was performed by Zhou *et al*. in 2014. The study concluded that GSTM1polymorphisms, but not GST T1 polymorphisms, are related to noise-induced hearing loss. There are at least two major differences between Zhou’s study and our study. (1) Zhou *et al*. evaluated the association of GST M1 and T1 polymorphisms only with NIHL. Five studies were included in their meta-analysis, with a total of (GST M1 and T1) 914/909 cases with NIHL and 885/876 controls. However, the purpose of our study was to identify the possible relationship between GST M1 and T1 polymorphisms and acquired SNHL, which includes three other kinds of SNHL in addition to NIHL, with a total of 1749 cases and 2018 controls included in our meta-analysis. (2) Zhou *et al*. concluded that GST M1 polymorphisms are related to NIHL, but in our study, we found opposite results, although the included studies and samples in the NIHL subgroup in our study were the same as those included in their study. The different results can be attributed to the different models used in the two studies. In Zhou’s study, the fixed-effects model was applied to calculate the pooled effect size even though significant heterogeneity (51%) was observed among the studies. Such a method is worth discussing.

Heterogeneity is a major problem that affects the reliability of the pooled effect size in meta-analysis. In our meta-analysis, heterogeneity was observed for both GSTM1 and GST T1. The results of the subgroup analysis according to the type of acquired SNHL and ethnicity showed that heterogeneity was much smaller in some subgroups, but it was strong in other groups, suggesting that some other factors besides the acquired SNHL type and ethnicity served as sources of heterogeneity. We also performed a meta-regression analysis (Supplementary Table S1) and found that the various sample sizes, different publication date and diverse Newcastle-Ottawa scale (NOS) scores in the included studies were not major source of heterogeneity either. Other factors that may influence heterogeneity are listed as follows: (1) the diagnostic criteria of hearing loss in each study are not completely consistent; (2) the matching methods of cases and controls in different studies are diverse; (3) diverse methods of GST M1 and T1genotype detection were used; (4) the quality of each study (NOS score) was not completely consistent; and (5) different age ranges were involved in the included samples in each study.

There are at least two limitations in this meta-analysis. (1) Several studies that possibly met the inclusion criteria did not include primary data, so the ORs with their 95% CIs could not be calculated. We attempted to contact the authors for more information, but we received no response. The results may be influenced by these missing studies. (2) Although 17 independent studies containing a total of 1749 cases with acquired SNHL and 2018 controls were included in this meta-analysis, the sample size is still limited, especially in the process of subgroup analysis. For the DIHL subgroup, most studies contained only 10–20 samples, and for the SSHL subgroup, only 2 articles met the inclusion criteria.

To our knowledge, this is the first meta-analysis to focus on the association of GST M1 and T1 polymorphisms with the susceptibility to acquired SNHL. The results of our meta-analysis suggested that GST M1 and T1 polymorphisms may not serve as susceptibility factors for acquired SNHL. Considering the limitations of our meta-analysis, further prospective studies with large sample size and additional studies (e.g. effect of this polymorphism on gene expression, haplotype analysis for GST polymorphism etc.) are needed to validate study findings.

## Methods

### Search strategy

A comprehensive literature search was performed in the following databases: (1) PubMed; (2) Web of Science; (3) EMBASE; (4) OVID; (5) CNKI Chinese database and (6) Wanfang Chinese database. The MeSH and free terms were all included in our search terms, which are listed as follows: “Glutathione s-transferase”, “Glutathione transferase”, “hearing impairment”, “hearing loss”, “ototoxicity” and “deafness”. Our search logic in the PubMed database is listed as follows: “((((“hearing”[MeSH Terms] OR “hearing”[All Fields]) OR (“ear, inner”[MeSH Terms] OR (“ear”[All Fields] AND “inner”[All Fields]) OR “inner ear”[All Fields] OR “cochlea”[All Fields] OR “cochlea”[MeSH Terms])) OR ototoxicity[All Fields]) OR (“audiology”[MeSH Terms] OR “audiology”[All Fields])) AND (((“glutathione transferase”[MeSH Terms] OR (“glutathione”[All Fields] AND “transferase”[All Fields]) OR “glutathione transferase”[All Fields] OR “glutathione s transferase”[All Fields]) OR (“Glutathione Transferase”[Mesh] AND “Glutathione S-Transferase pi”[Mesh] AND “glutathione S-transferase T1”[Supplementary Concept] AND “glutathione S-transferase M1”[Supplementary Concept])) OR (“glutathione transferase”[MeSH Terms] OR (“glutathione”[All Fields] AND “transferase”[All Fields]) OR “glutathione transferase”[All Fields])) AND “humans”[MeSH Terms]”.

All studies that we searched were published before November 20^th^, 2018. We also manually checked all articles listed in the reference lists of the retrieved literature.

### Inclusion criteria

Studies that met the following criteria were included: (1) independent studies investigating the relationship between GST M1 and T1 polymorphisms and the susceptibility to acquired SNHL and (2) studies including sufficient and definite original data (the genotype frequencies of GST M1 and T1 in the case and control groups) that could be used to calculate the OR with its 95% CI of each genotype. When duplicate publications were found, the data in the latest publication were used.

### Data extraction and Quality assessment

The data in the included studies were extracted by two investigators independently using the same “Data Extraction Form”. The information extracted from the included studies is listed as follows: first author’s name, publication year, country of origin, ethnicity, genotype detection methods, the type of SNHL, and the number of cases and controls. The quality of each included study was evaluated using the Newcastle-Ottawa scale (NOS). The studies with an NOS score ≥7 were considered high-quality studies. All disagreements in the process of study selection, data extraction and quality assessment were discussed and resolved by consensus.

### Meta-analysis

The association of GST M1 and T1 polymorphisms with acquired SNHL susceptibility was evaluated by the pooled OR and 95% CI.Statistical heterogeneity among the studies was measured with the I^2^ test. For a value of I^2^ < 30% and p > 0.1, a fixed-effects model was used to calculate the pooled ORs; otherwise, a random-effects model was used for a value of I^2^ ≥ 30%.Woolf’s method was applied to estimate the 95% CIs. We considered that there was statistical significance when the overall 95% CI did not include 1 and the p-value transformed from the Z score was less than 0.05. In addition, a subgroup analysis was performed according to the type of acquired SNHL and ethnicity. Sensitivity analysis was used to evaluate the stability of the pooled effect size. Publication bias was assessed by Egger’s test. Publication bias was considered for a p-value < 0.05 or if the 95% CI did not contain 0. All statistical analyses were performed using the Stata 13.1 software.

## Supplementary information

Results of meta-regression considering sample size, publication year and NOS scores of the included studies.

## Data Availability

All data generated or analyzed during this study are included in this published article (and its supplementary information files).
