# Supplementary material for: Association of Glutathione s-transferase M1 and T1 gene polymorphisms with the susceptibility to acquired sensorineural hearing loss: a systematic review and meta-analysis
Source: Sci Rep. 2019 Jan 29;9:833. doi: 10.1038/s41598-018-37386-w (PMC6351579; doi:10.1038/s41598-018-37386-w)
Supplement: Supplementary file 1 — Results of meta-regression considering sample size, publication year and NOS scores of the included studies. [file 41598_2018_37386_MOESM1_ESM.pdf]

Association of Glutathione s-transferase M1 and T1 gene polymorphisms with the susceptibility to acquired sensorineural hearing loss: a systematic review and meta-analysis

Shimin Zong[1+], Xue Zeng[2+], Yexiao Guan[1+], Tianyi Liu[1], Pan Luo[1], Fangmin Wan[1], Yanji Qu[1], Pei Chen[3], Hongjun Xiao[1*]

[1] Department of Otorhinolaryngology, Union Hospital, Tongji Medical College, Huazhong University of Science and Technology, Wuhan 430022, China

[2]Department of Obstetrics and Gynecology, Tongji Hospital, Tongji Medical College, Huazhong University of Science and Technology, Wuhan 430032, China

[3]Department of Otorhinolaryngology, Wuhan Integrated TCM and Western Medicine Hospital (Wuhan No. 1 hospital), Tongji Medical College, Huazhong University of Science and Technology, Wuhan 430022, China

[*]Corresponding. xhjent_whxh@hust.edu.cn (Hongjun Xiao)

[+]The authors contribute equally to this work.

	Gene name	Before meta-regression	After meta-regression	Variation
Tau - squared	GST M1	0.0901	0.06719	25.4%
	GST T1	0.1757	0.2011	-

Supplementary Table 1. Results of meta-regression considering sample size, publication year and NOS scores of the included studies. GST: glutathione s-transferase.
